# The Knockout of Enterobactin-Related Gene in *Pectobacterium atrosepticum* Results in Reduced Stress Resistance and Virulence towards the Primed Plants

**DOI:** 10.3390/ijms22179594

**Published:** 2021-09-04

**Authors:** Vladimir Gorshkov, Olga Parfirova, Olga Petrova, Natalia Gogoleva, Evgeny Kovtunov, Vladimir Vorob’ev, Yuri Gogolev

**Affiliations:** 1 Kazan Institute of Biochemistry and Biophysics, FRC Kazan Scientific Center of RAS, 420111 Kazan, Russia; olga.parfirova@kibb.knc.ru (O.P.); olga.petrova@kibb.knc.ru (O.P.); gogoleva@kibb.knc.ru (N.G.); kovtunovea@mail.ru (E.K.); vorobyev@kibb.knc.ru (V.V.); gogolev@kibb.knc.ru (Y.G.); 2 Institute of Fundamental Medicine and Biology, Kazan Federal University, 420008 Kazan, Russia

**Keywords:** siderophores, *Pectobacterium*, enterobactin, virulence factors, priming, salicylic acid, oxidative stress

## Abstract

Siderophores produced by microorganisms to scavenge iron from the environment have been shown to contribute to virulence and/or stress resistance of some plant pathogenic bacteria. Phytopathogenic bacteria of *Pectobacterium* genus possess genes for the synthesis of siderophore enterobactin, which role in plant-pathogen interactions has not been elucidated. In the present study we characterized the phenotype of the mutant strain of *Pba* deficient for the enterobactin-biosynthetic gene *entA*. We showed that enterobactin may be considered as a conditionally beneficial virulence factor of *Pba*. The *entA* knockout did not reduce *Pba* virulence on non-primed plants; however, salicylic acid-primed plants were more resistant to Δ*entA* mutant than to the wild type *Pba*. The reduced virulence of Δ*entA* mutant towards the primed plants is likely explained by its compromised resistance to oxidative stress.

## 1. Introduction

*Pectobacterium* and *Dickeya* species belonging to soft rot *Pectobacteriacea* (SRP) cause devastating plant diseases [1]. Their main virulence determinants are plant cell wall degrading enzymes (PCWDEs) [2]. However, PCWDEs alone are not sufficient for SRP to be able to cause disease, and additional virulence factors were described for these pathogens. For example, for *Pectobacterium* species, Svx protein [3], Nip protein [4], type 3 secretion system (T3SS) [5,6], T6SS [7,8], coronafacic acid [9,10,11] were shown to be required for full virulence. For *Dickeya* species, in addition to PCWDEs, siderophores were described as major virulence factors [12].

Siderophores are small metabolites (usually less than 1 kDa) that scavenge iron from mineral and organic substrates due to high iron-affinity and deliver it to cells via specific receptors [13,14]. Iron is an essential nutrient for fundamental metabolic processes in living organisms. Owing to its capacity to undergo reversible changes in its oxidation state, iron is a privileged cofactor for many proteins mediating electron transfer and redox reactions. This metal is required for pathogens to manifest their virulence potential. Even though iron is the fourth most abundant element in the Earth’s crust, it is poorly bioavailable at alkaline or neutral pH because of the low solubility of ferric hydroxides prevailing in aerobic soils [15,16]. Siderophores serve as iron carriers in bacteria and other organisms. Iron-free siderophores are exported from the microbial cell, while the ferric-siderophore complexes can be taken up via specific outer-membrane transporters associated with the TonB complex. Inside the microbial cell, iron within siderophore is reduced, losing its affinity for the siderophore, and Fe^2+^-ions are then distributed to iron-containing molecules [12]. In addition to serving as iron carriers, siderophores can provide non-iron metal transport, toxic metal sequestration; they function as signaling molecules and protect cells from oxidative stress [13]. Siderophores play important roles in plant-microbe interactions. Siderophores of some plant-growth-promoting rhizobacteria were shown to trigger the induced systemic resistance (ISR) in plants [12,17], while siderophores of some plant pathogens can act as virulence factors.

Among plant pathogens, siderophores were described in a range of fungi [18] as well as in bacteria *Erwinia amylovora* (deferrioxamine, DFO) [19], *Pseudomonas syringae* (pyoverdine) [20], *Agrobacterium tumefaciens* (agrobactin) [21] and *Dickeya dadantii* (previously *Erwinia chrysanthemi*) (chrysobactin and achromobactin) [22,23]. The latter species became a classical object of studying the role of siderophores in plant-pathogen interactions.

Chrysobactin (CB)—a monocatecholate-type siderophore and achromobactin—citrate derived compound are produced by *D. dadantii* in a sequential manner; achromobactin is produced before CB and its production decreases as that of CB increases. Both siderophores are necessary for successful infection and systemic spreading of soft rot symptoms [24,25]. CB was shown to outcompete host plant ferritins in iron acquisition and thus steal host plant iron [26,27]. In addition, iron-free (but not iron-bound) siderophores, including CB, as well as synthetic iron chelators by causing complex disturbance of metal homeostasis trigger the plant responses related to immunity activation and iron assimilation [12,26,28,29]. CB and some other siderophores activate salicylic acid (SA)-mediated defenses, including ROS accumulation and callose deposition, acting as elicitors [26,28,29]. In addition, CB and DFO induce a leaf-to-root iron deficiency signal and activate iron uptake by the roots [28,29,30]. Activation of iron uptake is usually considered as a defense mechanism, since iron is necessary for ROS generation and other immunity-related processes [28]. Preinfectional treatment of plants with siderophores (DFO, pseudobactine) increased their resistance to *Pseudomonas syringae*, *Ralstonia solanacearum* and *Colletotrichum graminicola* [26,31,32]. However, the treatment of plants with CB increased susceptibility to *D. dadantii* [33]. Moreover, when plants were grown under iron-deficiency, their resistance to *D. dadantii* was increased despite the fact that the immunity-related parameters of the host plants (ROS level and callose deposition) were reduced [33]. This means that CB-induced activation of iron uptake by the host plant is beneficial for *D. dadantii*, and CB is used by this pathogen for both iron acquisition and manipulating with host plant reactions. Thus, the role of siderophores in the modulation of plant responses is multifaceted and depends on the strategy of plant-pathogen interactions.

In spite of the fact that *Pectobacterium* species are closely related to *Dickeya* species and both genera utilize similar strategies of host plant colonization, pectobacteria do not produce CB or achromobactin. Instead, pectobacteria possess a cluster of genes for biosynthesis of enterobactin [9]—a siderophore widely represented among *Enterobacterales* [34]. In our previous study we have shown that the cluster of enterobactin-related genes as well as tonB genes necessary for the siderophore transport were among the most upregulated *Pba* genes during symptomatic and asymptomatic plant colonization [10]. However, the role of enterobactin in *Pectobacterium*-plant interaction has not been studied to date. The aim of our study was to understand the role of enterobactin in *Pba* virulence and resistance. Herewith, we hypothesized that enterobactin can contribute to *Pba* virulence and/or its resistance to different stressors including the defense reactions of the colonized plant.

## 2. Results

### 2.1. Primary Characteristics of Enterobactin-Deficient Pba Mutant

**Figure 1:**
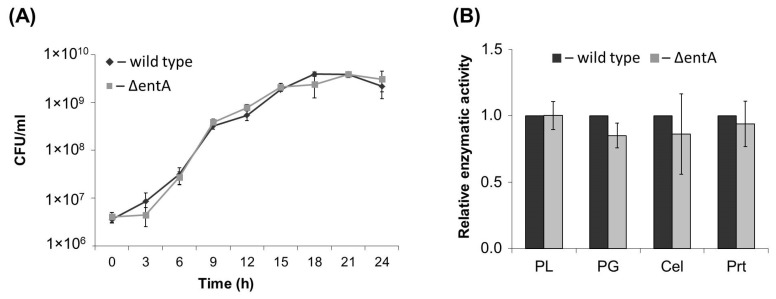
Growth curves (**A**) and activities of extracellular enzymes (**B**) of the wild type (dark grey) and Δ*entA* mutant (light grey) of *Pectobacterium atrosepticum* SCRI1043. PL—pectate lyase, PG—polygalacturonase, Cel—cellulase, Prt—protease. The enzymatic activities were determined in the culture supernatants after one day of bacteria cultivation. The presented values are means ± SD of three biological replicates. The activity levels of the wild type were equated to one. No significant differences in the assessed parameters were revealed between the wild type and Δ*entA* mutant (Mann-Whitney two-sided test, *p* < 0.05).

The obtained Δ*entA* mutant did not differ in its growth in vitro from the wild type strain (Figure 1A). The mutant produced similar to the wild type levels of extracellular pectate lyase, polygalacturonase, cellulase and protease (Figure 1B). Taken together, the target mutation did not cause shifts in cultural characteristics and production of major virulence determinants of *Pba*.

### 2.2. Virulence of ΔentA Mutant

**Figure 2:**
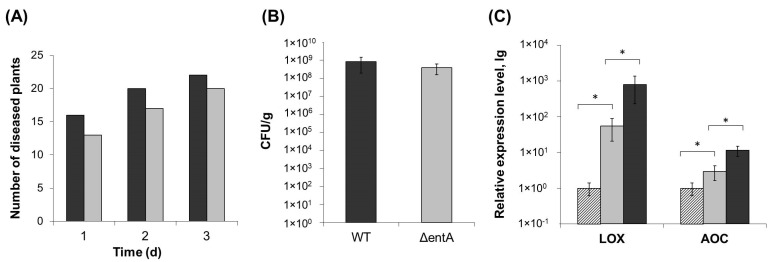
Virulence of Δ*entA* mutant of *Pectobacterium atrosepticum* SCRI1043. (**A**) Number of tobacco plants displaying maceration symptoms after infection with the wild type (dark grey) or Δ*entA* mutant (light grey) of *P. atrosepticum*. (**B**) Bacterial colony forming unit (CFU) titer in the tobacco plants infected with the wild type (dark grey) or Δ*entA* mutant (light grey) of *P. atrosepticum*. (**C**) Expression levels of genes LOX (lipoxygenase) and AOC (allen oxide cyclase) in non-infected plants (hashed column) and infected with the wild type (dark grey) or Δ*entA* mutant (light grey) of *P. atrosepticum*. The expression levels and CFU titers were determined one day post inoculation. The presented values in B and C are means ± SD of five biological replicates. Asterisks (*) show the significance of difference (Mann–Whitney two-sided test, *p* < 0.05).

To check if *entA* gene knockout affected *Pba* virulence, tobacco plants were infected with the wild type or mutant strain. Almost 90% of plants infected by the wild type displayed maceration symptoms expressed in tissue softening and disintegration 3 days post inoculation. Δ*entA* mutant-infected plants showed similar symptoms, and only 10% less plants had macerated zones compared to plants infected by the wild type (Figure 2A). Similar results were obtained on the infected potato plants (data not shown). The titer of bacterial cells in the diseased tobacco plants infected with either the wild type or Δ*entA* mutant differed only around two times that was not statistically significant (Figure 2B). Since the marker of plant susceptibility to *Pba* is the expression level of jasmonic acid-regulated genes [10,35], the levels of gene transcripts of lipoxygenase (LOX2) and allene oxide cyclase (AOC) was compared in tobacco plants infected with either the wild type or Δ*entA* mutant, on which symptoms were equally manifested. LOX2 and AOC genes were induced in plants infected with the wild type as well as mutant strain compared to control non-infected plants (Figure 2C). However, the level of induction was lower in the plants infected by the Δ*entA* mutant. Thus, the knockout of *entA* gene did not make significant contribution to virulence properties of *Pba*. However, the expression level of jasmonic acid-regulated genes, the markers of *Pba*-caused disease, was decreased in Δ*entA* mutant-infected plants compared to the wild type-infected ones.

### 2.3. Stress Resistance of ΔentA Mutant

**Figure 3:**
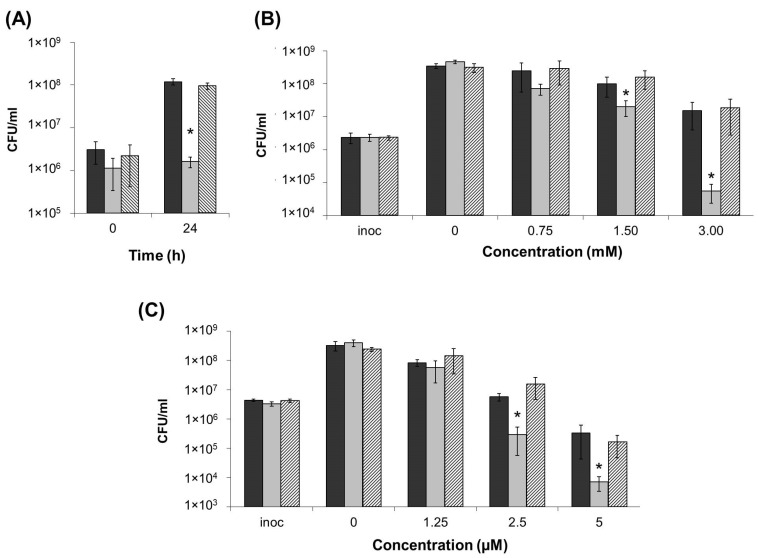
Resistance of the wild type (dark grey), Δ*entA* mutant (light grey) and Δ*entA* complementation mutant (hashed column) of *Pectobacterium atrosepticum* SCRI1043 to iron depletion (20 µM Na-EDTA) (**A**), oxidative stress (H_2_O_2_) (**B**) and heavy metal (CuSO_4_) (**C**). Cells were cultured 24 h before plating for CFU titer analysis. The presented values are means ± SD of three biological replicates of one of three representative experiments. Inoc—inoculation titer. Asterisks (*) show the significance of difference (Mann–Whitney two-sided test, *p* < 0.05).

Since siderophores were shown to contribute not only to virulence, but also to resistance to some stressors (foremost oxidative stress and heavy metals) [14,34,36,37,38,39], we compared the resistance of the wild type and Δ*entA* mutant as well as the complementation mutant carrying the lost *entA* gene within a recombinant plasmid. Herewith, the ability of bacterial strains to grow in iron-depleted medium (in the presence of 20 µM Na-EDTA) or in presence of hydrogen peroxide or CuSO_4_ was assessed. In contrast to the wild type and complementation mutant, Δ*entA* mutant was unable to grow in the presence of 20 µM Na-EDTA (Figure 3A).

During 24 h of cultivation at 3 mM concentration of H_2_O_2_, the cell titer of the wild type and complementation mutant increased 7–8-fold, while that of the mutant strain decreased by more than 40-fold (Figure 3B). At 1.5 mM of H_2_O_2_, all three strains were able to grow, but herewith, the cell titers in the wild type and complementation mutant cultures were 3–4-fold higher than that in the cultures of Δ*entA* mutant. At lower H_2_O_2_ concentrations, the differences between the wild type and mutant strains were insignificant. The CFU number decreased at 5 µM of CuSO_4_ in the cultures of all three strains compared to the inoculation titer, however, much greater reduction was observed for the Δ*entA* mutant strain compared to the wild type and complementation mutant (Figure 3C). At 2.5 µM CuSO_4_, the CFU titer of the wild type and complementation mutant increased slightly compared to the inoculation titer, while the CFU titer of the mutant strain decreased more than 10-times. At lower CuSO_4_ concentrations, the differences between the wild type and mutant strains were insignificant. Thus, the knockout of *entA* gene results in the decreased stress resistance of *Pba*.

### 2.4. Virulence of ΔentA Mutant towards Salicylic Acid-Primed Plants

**Figure 4:**
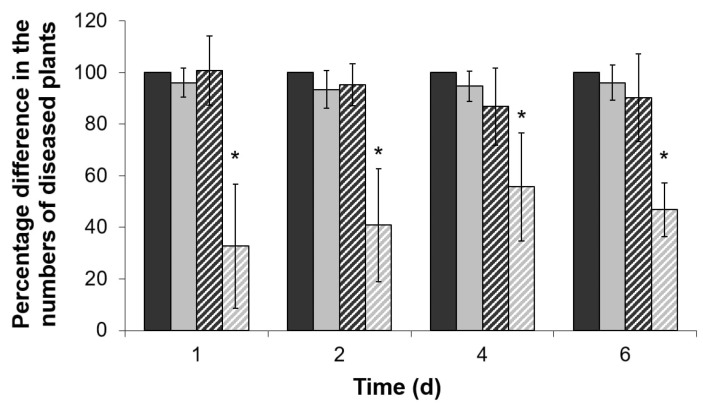
The disease incidents caused by the wild type *Pectobacterium atrosepticum* SCRI1043 (dark gray) or its Δ*entA* mutant (light grey) on mock-treated (solid columns) or 0.2 mM salicylic acid-treated (hashed columns) tobacco plants. The number of the diseased plants in mock-treated group infected by the wild type was equated to 100% in each time point. The presented values are means ±SD of four independent experiments performed at 15–25 biological replicates each. Asterisks (*) show the significance of difference (Mann–Whitney two-sided test, *p* < 0.05).

Even though the Δ*entA* mutant strain did not display reduced virulence compared to the wild type in the experiments described above, its increased stress susceptibility might impede the disease manifestation in the primed plants. To test this hypothesis, tobacco plants were pretreated with 0.2 mM salicylic acid (SA). This concentration was shown to be below the one (1 mM) that led to a significant reduction of the disease development caused by the wild type *Pba* (data not shown). Tobacco plants were mock- or SA-treated 24 h before the infection with either the wild type or Δ*entA* mutant. The number of plants with maceration symptoms was scored 1, 2, 4 and 6 days post inoculation. The number of the diseased plants in the mock-treated wild type-infected plant group (assign as a control group) was equated to 100% at each time point. The disease incidence rate in the SA-treated wild type-infected group and mock-treated Δ*entA* mutant-infected group did not differ significantly from the control group, but herewith, the disease incidence rate in the SA-treated Δ*entA* mutant-infected group was 2–3-times lower (Figure 4). Thus, enterobactin deficiency impeded *Pba* to cause the disease on the SA-primed plants, but not on non-primed plants.

**Figure 5:**
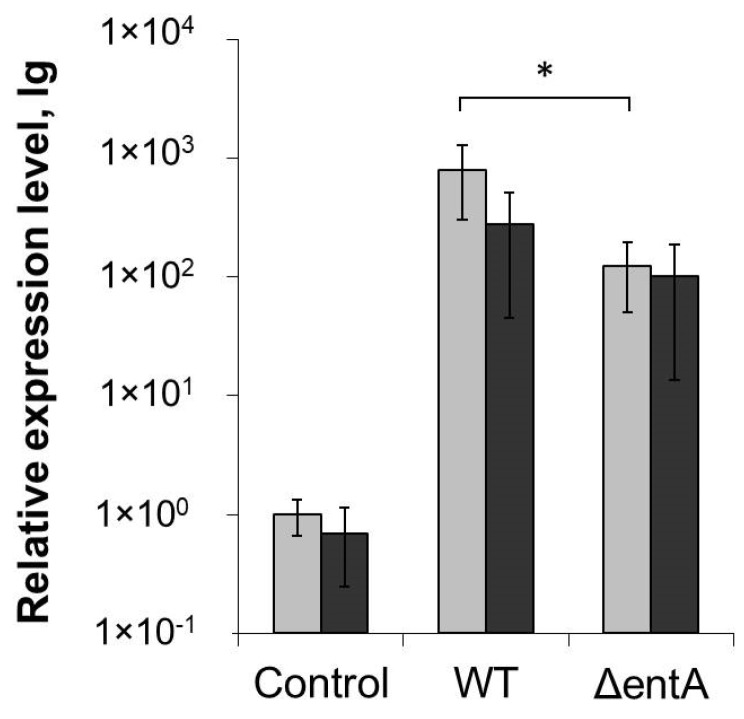
Expression level of LOX2 (lipoxygenase) gene in non-primed (light grey) and salicylic acid-primed (dark grey) tobacco plants non-infected (control) or infected with the wild type (WT) or Δ*entA* mutant (Δ*entA*) of *Pectobacterium atrosepticum* SCRI1043. The expression levels were determined one day post inoculation. The presented values are means ± SD of four biological replicates. Asterisk (*) shows the significance of difference (Mann–Whitney two-sided test, *p* < 0.05).

The LOX2 gene expression (the marker of plant susceptibility to *Pba*) was induced in the wild type *Pba*- and Δ*entA* mutant-infected plants during the disease progression irrespective of whether the plants were treated with SA or not (Figure 5). However, the level of induction was slightly (non-significantly) lower in the SA-treated plants compared to non-treated ones. Herewith, the LOX2 gene expression level was greater in the non-treated wild type *Pba*-infected plants compared to that in the non-treated Δ*entA* mutant-infected plants (as also described above). However, in the SA-treated plants, the expression level of this gene did not differ significantly between the plants infected with the wild type and Δ*entA* mutant (Figure 5). The bacterial cell titer in planta also did not differ in the non-treated and SA-treated plants infected by the wild type *Pba* and Δ*entA* mutant (data not shown).

**Figure 6:**
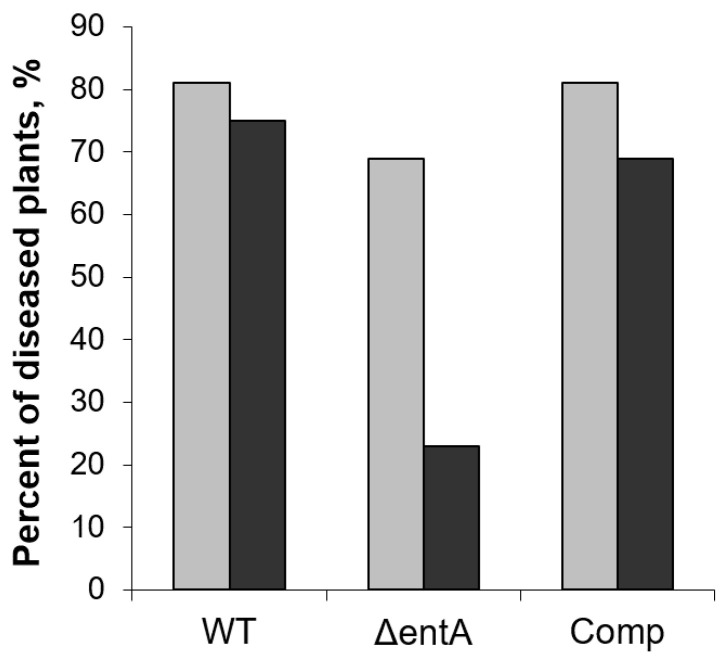
The percent of non-primed (light grey) and salicylic acid-primed (dark grey) tobacco plants showing disease symptoms after infection with the wild type (WT), or Δ*entA* mutant (Δ*entA*), or Δ*entA* complementation mutant (Comp) of *Pectobacterium atrosepticum* SCRI1043. The priming was performed one day before the inoculation. The symptoms were analyzed three days post infection.

Then we analyzed whether the complementation of Δ*entA* mutation restored the virulence towards the SA-primed plants. In this case, 69 and 81% of non-primed plants displayed disease symptoms three days after inoculation with Δ*entA* mutant and the complementation mutant, respectively. Herewith, in 23% and 69% of the SA-primed plants, the disease symptoms were developed after infection with Δ*entA* mutant and the complementation mutant, respectively (Figure 6). This means that the complementation of Δ*entA* mutation restored the virulence towards the SA-primed plants.

### 2.5. H_2_O_2_-Level in Infected and Non-Infected Tobacco Plants Pretreated or Not with Salicylic Acid

To check whether the SA-priming of plants (resulting in the increased plant resistance to Δ*entA* mutant but not to the wild type *Pba*) influenced the level of ROS and whether the ROS accumulation was different in plants (SA-treated and non-treated) infected by the wild type *Pba*, or Δ*entA* mutant, or complementation mutant, the level of hydrogen peroxide was measured. Herewith, among the plants infected with each of the strains, plants that displayed disease symptoms and plants that remained symptomless after the infection were analyzed separately.

**Figure 7:**
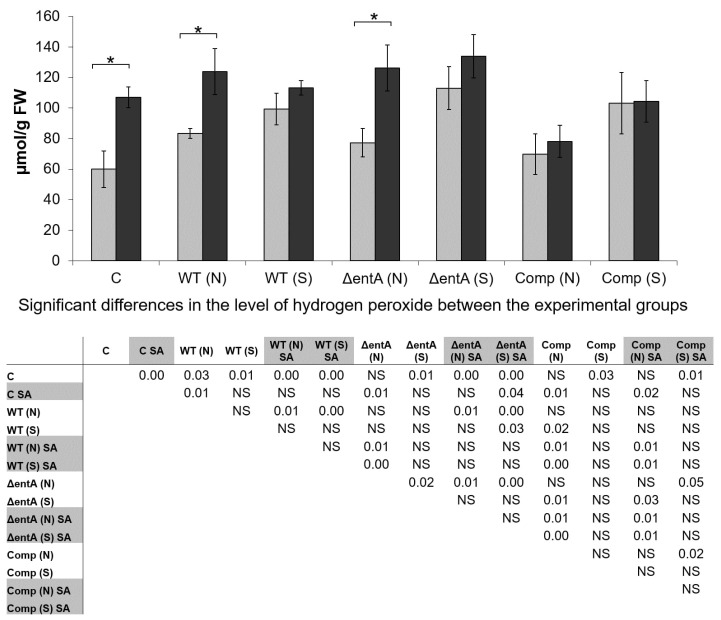
The level of hydrogen peroxide in non-primed (light grey) and salicylic acid-primed (dark grey) tobacco plants non-infected control (C) or infected with the wild type (WT), or Δ*entA* mutant (Δ*entA*), or Δ*entA* complementation mutant (Comp) of *Pectobacterium atrosepticum* SCRI1043. Plants with symptoms (S) and with no symptoms (N) were analyzed differentially within all experimental groups (WT, Δ*entA*, Comp) of infected plants. The priming was performed one day before the inoculation. Hydrogen peroxide was determined one day post inoculation. The presented values are means ± SD of three biological replicates. Asterisks (*) show the significance of difference (Mann–Whitney two-sided test, *p* < 0.05) between non-primed and salicylic acid (SA)-primed plants within a single variant (C, WT, Δ*entA*, Comp). The table located under the diagram shows significant differences between all experimental groups; (SA)-primed variants are marked by grey in the table. NS—non-significant. FW—fresh weight.

The SA-treatment resulted in the increased H_2_O_2_-level in control non-infected plants compared that in non-treated plants (Figure 7). Symptomatic infections in both the SA-treated and non-treated plants, irrespective of the strain that caused them (wild type *Pba*, or Δ*entA* mutant, or complementation mutant), were associated with the increased H_2_O_2_-level compared to that in non-treated non-infected plants; and herewith, the SA-treatment did not increase the H_2_O_2_-level in infected plants compared to that in non-treated infected plants.

In symptomless plants infected by the wild type *Pba*, the level of H_2_O_2_ was also increased compared to control; herewith, the H_2_O_2_ level did not differ in symptomless plants infected by different strains. During symptomatic infection caused by Δ*entA* mutant, the level of H_2_O_2_ was higher than that in symptomless plants infected by the same strain. Mean values of H_2_O_2_-level during symptomatic infections caused by two other strains (wild type *Pba* and complementation mutant) were also higher than during the asymptomatic ones although these differences were not statistically significant (Figure 7). During symptomatic infections caused by all three strains, the H_2_O_2_ levels did not differ in SA-primed and non-primed plants. However, in symptomless plants infected by the wild type *Pba* or Δ*entA* mutant, the levels of H_2_O_2_ were greater if the plants were SA-primed.

The level of H_2_O_2_ did not differ in non-primed plants infected by different strains during symptomatic infections, and the same was true for the non-primed symptomless plants infected by different strains. As for the SA-primed plants, their H_2_O_2_ levels did not differ during both symptomatic and asymptomatic infections caused by the wild type and Δ*entA* mutant. However, in the SA-primed symptomless plants infected by the complementation mutant, the level of H_2_O_2_ was lower than that in the SA-primed symptomless plants infected by the wild type and Δ*entA* mutant; a similar tendency was observed for symptomatic infection, however the differences were statistically insignificant (Figure 7).

Thus, the SA-priming leads to the increase in ROS level in tobacco plants. In turn, the increased ROS level within the SA-primed plants can prevent the manifestation of full virulence of Δ*entA* mutant due to its high ROS-vulnerability. In addition, given the decreased H_2_O_2_-level in the complementation mutant-infected SA-primed plants (compared to the wild type *Pba*- and Δ*entA* mutant-infected SA-primed plants) (Figure 7) as well as the increased expression level of *entA* gene within the recombinant plasmid (in the complementation strain) compared to its expression within the bacterial chromosome (in the wild type strain) (data not shown) it may be presumed that enterobactin can be involved in the detoxification of ROS within the infected plants.

## 3. Discussion

One of the genera of soft rot *Pectobacteriaceae*, *Dickeya*, was unequivocally shown to use siderophores (chrysobactin and achromobactin) to cause disease in host plants. Herewith, *Dickeya* siderophores act as both iron carriers and inducers of host reactions related to iron assimilation, which is necessary for the disease development [12,26,28,29,33]. As for the second genus of soft rot *Pectobacteriaceae*, *Pectobacterium*, siderophores have not been characterized. *Pectobacterium* species do not have genes related to chrysobactin and achromobactin synthesis but possess a cluster of the enterobactin-biosynthetic genes, which expression is highly induced during plant colonization [9,10]. In the present study we characterized the phenotype of the mutant strain of *Pba* deficient for one of the enterobactin-biosynthetic genes *entA*. We have shown that the knockout of *entA* gene causes only tiny, if any, effect on the disease development. The mutant produced as much extracellular enzymes (pectate lyase, polygalacturonase, cellulase, protease) as the wild type did and the symptoms of disease did not differ on plants infected with the wild type strain and Δ*entA* mutant. Only low non-significant reduction in the number of the diseased plants was observed for the Δ*entA* mutant-infected plants compared to the wild type-infected plants. Herewith, plant genes of the jasmonate-related pathway, which expression may serve as a marker of *Pba*-caused disease, were upregulated in the Δ*entA* mutant-infected tobacco plants significantly less than in the wild type-infected plants. Considering these results, it can be preliminary concluded that enterobactin does not make a significant contribution to *Pba* virulence. This is consistent with the fact that siderophore-deficient mutants of some phytopathogenic bacteria fully retained virulence [19,40,41,42].

Differences in the consequences of siderophore-deficiency for closely related phytopathogens, *Pectobacterium* and *Dickeya*, can be explained by the following. First, *D. dadantii*, produces a specific pectate lyase, named PelN, that requires iron as a co-factor for its enzymatic activity in contrasts to iron-independent pectate lyases of pectobacteria [43]. Therefore, *Pectobacterium* and *Dickeya* may require different levels of iron to maintain their major virulence factors in active state. Second, siderophores are not the only way of iron acquisition [2] and alternative iron-acquisition systems may compensate siderophore deficiency in *Pectobacterium* species better than in *Dickeya* species. For example, *Pectobacterium* species have a transport system (FecABCD) for ferric citrate, which is formed in plant tissues and provides a long-distance transport of iron [15].

In turn, the enterobactin-deficiency caused a significant reduction in *Pba* stress resistance, including oxidative stress. It is known, that siderophores, including enterobactin, act as antioxidants and contribute to bacterial resistance to ROS as well as heavy metals [14,34,36,37,38,39]. Taking this into account, we have hypothesized that we did not observe the reduction of Δ*entA* mutant virulence compared to the wild type because the virulence test was carried out under infection-promoting conditions. We presumed that the Δ*entA* mutant virulence could be impaired if tested on the primed plants. The priming state is characterized by preinduced immunity, which enables to respond more rapidly and effectively to pathogen invasion. To test this hypothesis, we used SA. This phytohormone is known to contribute to oxidative stress-related plant defenses [44] and is shown to confer resistance to SRP [45,46]. We used 0.2 mM SA, a concentration that did not reduce significantly the disease development caused by the wild type. However, this SA concentration was sufficient to significantly reduce disease incidents caused by Δ*entA* mutant strain. The SA-priming of plants led to an increase in their H_2_O_2_-level, to which Δ*entA* mutant displayed compromised resistance in vitro compared to the wild type. Thus, enterobactin is likely to contribute to the resistance of *Pba* to the host plant-produced ROS. However, the increased level of plant-produced H_2_O_2_ did not influence the growth of the mutant strain since the CFU titers of the Δ*entA* mutant and the wild type did not differ significantly in the SA-primed plants. This means that the reduced virulence of Δ*entA* mutant towards the SA-primed plants was not related to its reduced growth in planta. The observed influence of the target mutation on *Pba* stress resistance and virulence towards the SA-primed plants was not a result of a polar effect of the mutation. The complementation of this mutation restored the wild type phenotype in Δ*entA* mutant carrying the *entA* gene within a recombinant plasmid. In addition, the gene located downstream of the *entA* gene (ECA0482, encoding non-ribosomal peptide synthetase) was expressed at similar level in the wild type and Δ*entA* mutant (data not shown).

Our results show that in addition to its role in stress resistance, enterobactin may be considered as a conditionally beneficial virulence factor of *Pba*. Its effect on virulence is undetectable if non-primed plants are tested. However, in the SA-primed plants, enterobactin significantly contributes to *Pba* virulence. Given that under natural conditions plants are continuously exposed to stress factors and thus are in primed state, the significant upregulation of enterobactin related genes [10] evidently gives strong benefit to *Pba* in plant colonization. Interestingly, another siderophore, DFO, can also be regarded as a conditionally beneficial virulence factor of *E. amylovora.* DFO appeared to be unnecessary for the bacteria to cause the disease on apple seedlings but was explicitly required for the colonization of apple flowers and ROS resistance [19]. This means that the conclusions that siderophores do not make contribution to the virulence of some plant pathogenic bacteria [19,40,41,42] may be premature and a number of experimental conditions should be tested to judge about the role of a gene or metabolite in virulence.

Taken together, enterobactin contributes to *Pba* stress resistance. Enterobatin is also necessary for *Pba* to cause disease in that plant, whose immune system is in the primed state. Herewith, if the plant is non-primed, the enterobactin does not make significant contribution to *Pba* virulence.

## 4. Materials and Methods

### 4.1. Bacterial Strains, Media and Culture Conditions

*Pectobacterium atrosepticum* SCRI1043 (*Pba*) (ATCC BAA-672) was grown in Luria-Bertani (LB) medium on a rotary shaker (180 rpm) at 28 °C. The Δ*entA* mutant strain was grown in the presence of kanamycin (20 μg/mL) and the complementation mutant was grown in the presence of kanamycin (20 μg/mL) and ampicillin (200 μg/mL). The CFU titer was determined by the plating of serial 10-fold dilutions of the cultures onto 1.5% LB agar. For stress tolerance assay, minimal medium D5 (0.1 mM Na-K phosphate buffer (pH 7.5), 1.0 g L^−1^ NH_4_Cl, 0.3 g L^−1^ MgSO_4_·H_2_O, 2.0 g L^−1^ sucrose) was used. For the extracellular enzymatic activity assays, minimal medium D5 was supplemented by 2.0 g L^−1^ pectin (FLUKA) instead of sucrose.

### 4.2. Construction of entA Deletion Mutant

The *entA* deletion mutant (Δ*entA*) was constructed by the method described by Kaniga et al. (1991). The target gene *entA* (ECA0481 locus) together with the adjacent regions (approximately 1000 bp up- and downstream of *entA* ORF) were amplified by PCR with primers **up***entA*F and **dn***entA*R (Table S1; Figure S1) using Q5 high-fidelity DNA polymerase (NEB, Ipswich, MA, USA). The amplified PCR fragment was cloned into the bacterial cloning vector system pGEM-T Easy (Promega, Madison, WI, USA). The obtained plasmid (pGEM:*entA*) was introduced into *E. coli* NovaBlue by chemical transformation. Transformants carrying the recombinant plasmid were screened by ampicillin resistance and further verified by PCR using plasmid specific primers for the T7 and SP6 polymerase promoters, which flank the multiple cloning regions of pGEM-T Easy.

To replace *entA* ORF with the Km^R^ cassette, a part of pGEM:*entA* plasmid (including ~1000 bp regions up- and downstream of *entA* ORF but not *entA* ORF itself) was amplified with primers **dn***entA*KmF and **up***entA*KmR (Table S1; Figure S1), whose 5′-ends were complementary to the end regions of Km^R^ cassette. The amplified PCR fragment was treated with restriction endonuclease DpnI to remove the original methylated plasmid and then purified using DNA cleanup kit (NEB, USA). Km^R^ cassette was amplified from pKD4 plasmid with primers **Km***entA*F and **Km***entA*R, whose 5′-ends were complementary to *Pba* DNA regions adjacent to *entA* ORF. Two obtained PCR fragments (corresponding to pGEM plasmid with ~1000 bp regions up- and downstream of *entA* ORF and to Km^R^ cassette) were joined by circular polymerase extension cloning method [47] (Figure S1). The obtained plasmid (pGEM:Δ*entA*;Km^R^) was introduced into *E. coli* NovaBlue by chemical transformation. The mutant locus was confirmed by DNA sequencing.

The mutant locus (containing the Km^R^ cassette and ~1000 bp regions up- and downstream of *entA* ORF) was amplified with primers **up***entA*F and **dn***entA*R (Table S1; Figure S1) and ligated (T4 ligase, NEB, USA) into the SmaI-digested (NEB, USA) suicide vector pKNG101 to generate the recombinant plasmid containing the allelic exchange cassette for the target locus. The obtained plasmid (pKNG101:Δ*entA*;Km^R^) was introduced into *E. coli* cc118 by electroporation. The transfer of pKNG101:Δ*entA*;Km^R^ plasmid from *E. coli* cc118 into *Pba* was achieved by triparental mating using *E. coli* HH26 as a helper strain. The clones, in which pKNG101:Δ*entA*;Km^R^ plasmid was integrated into the chromosome by a single crossover event, were selected by streptomycin and kanamycin resistance. The clones, in which the second crossover event led to the replacement of the target locus with the mutant one and the donor plasmid was eliminated, were selected on M9 agar medium containing 10% sucrose. Then, the clones were tested for the sensitivity to streptomycin. Clones without streptomycin resistance were analyzed by PCR with primers **Cheсk***entA*F and **Check***entA*R to identify Δ*entA* mutants.

### 4.3. Construction of the Complementation ΔentA Mutant Strain Carring the entA Gene within the Plasmid

The *entA* is the fifth gene within the operon that contains seven genes (*entCEBFA* and two non-ribosomal peptide synthetases). To construct the complementation plasmid, the target gene *entA* as well as 343 bp promoter region of the operon and 153 bp terminator region were amplified by PCR with primers **prom***entA*F and **entA***term*R, promF and **prom***entA*R, **entA***term*F and termR, respectively (Table S1), using Q5 high-fidelity DNA polymerase (NEB, USA). The amplified PCR fragments were assembled by overlapping PCR. The assembled fragment was cloned into the bacterial cloning vector system pGEM-T Easy (Promega, USA). The obtained plasmid (pGEM:*entA*; complementation construct) was introduced into *E. coli* NovaBlue by chemical transformation. Transformants carrying the recombinant plasmid were screened by ampicillin resistance and further verified by PCR using plasmid specific primers for the T7 and SP6 polymerase promoters, which flank the multiple cloning regions of pGEM-T Easy. The correct assembly was confirmed by DNA sequencing.

The obtained plasmid (pGEM:*entA*; complementation construct; Amp^R^) was introduced into *Pba* Δ*entA* (Km+) strain by electroporation. Clones with ampicillin and kanamycin resistance were analyzed by PCR with primers **Km**entAF/**Km**entAR and entAF/entAR to identify Δ*entA* mutants with the complementation construct.

### 4.4. Plant Cultivation and Infection

*Nicotiana tabacum* cv. Petit Havana SR1 plants were grown axenically in test tubes placed in a growth chamber with a 16-h light/8-h dark cycle photoperiod. Seeds were surface-sterilized using diluted bleach (0.8% of active chlorine) and 1% sodium dodecyl sulfate for 30 min, washed seven times with sterile distilled water, then transferred to Murashige and Skoog medium (MS) in Petri dishes. In this case, 10-day-old seedlings were transferred to individual flasks containing MS. Four to five weeks after planting, plants were infected with *Pba*, or Δ*entA* mutant, complementation mutant. For plant inoculation, bacteria were grown until the early stationary phase (~2 × 10^9^ colony-forming units, CFU mL^−1^), then washed with sterile 10 mM MgSO_4_ and resuspended in the same solution up to a density of ~2 × 10^7^ CFU mL^−1^. Sterile 10 mM MgSO_4_ or bacterial suspensions containing ~2 × 10^5^ cells were placed as 10 μL drops into the bosoms of the leaves in the middle part of the stems using sterile pipette tips and slight scratches were made simultaneously. In the experiments with plant priming, the plants were treated by pulverization with ~350 µL per plant of a solution of 0.2 mM salicylic acid or water 24 h before inoculation.

Virus-free potato (*Solanum tuberosum* cv. Condor) plants were vegetatively propagated under sterile conditions. Apical parts (2 cm length) of sterile plants were rooted aseptically in vermiculite. Then, the plants were grown in 300 mL plastic pots on commercial soil (Peter Peat, Dzerzhinsky, Russia) with a 16-h light/8-h dark cycle photoperiod at 20 °C. Five weeks after planting into the soil, plants were infected by *Pba* or Δ*entA* mutant by injecting 20 µL of the bacterial suspension (~2 × 10^7^ CFU mL^−1^) into the middle part of a stem.

### 4.5. Stress Tolerance Assay

To compare stress resistance of the wild type *Pba*, Δ*entA* mutant and complementation mutant, bacteria were cultured in D5 medium supplemented with 2.0 g L^−1^ sucrose in the absence or in the presence of Na-EDTA (20 µM), H_2_O_2_ (0.7, 1.5 or 3.0 mM) or CuSO_4_ (1.25, 2.5, 5 μM). After 24 h of cultivation, suspensions were plated onto 1.5% LB agar as serial 10-fold dilutions. The plates were incubated at 28 °C for 2 days before the CFUs were counted.

### 4.6. Enzymatic Activity Assays

Pectate lyase activity was determined by measuring the degradation of polygalacturonic acid (PGA) into unsaturated products [48]. First, 435 μL of 0.25% PGA (Sigma, Ronkonkoma, NY, USA) in 50 mM Tris-HCl buffer (pH 8.5) was mixed with 50 μL of 10 mM CaCl_2_ and 50 μL of the cultural supernatant at 37 °C. The accumulation of the unsaturated products was measured at 234 nm. One unit of pectate lyase activity was defined as the amount of enzyme releasing 1 μmol of unsaturated products/min per 10^9^ bacterial cells.

Cellulase and polygalacturonase activities were determined by measuring the reducing sugars released after the enzymatic hydrolysis of the corresponding substrates. The reducing sugars were measured using 3,5-dinitrosalicylic acid (DNS reagent) (Sigma, USA) at 540 nm [49]. Cellulase (endoglucanase) activity was determined using carboxymethyl cellulose as a substrate (Sigma, USA). Herewith, 250 μL of the cultural supernatant were mixed with 250 μL of 2% carboxymethyl cellulose in 100 mM citrate buffer (pH 5.5) and incubated 30 min at 50 °C [50]. For the determination of polygalacturonase activity, 180 μL of PGA (5 mg/mL) in 50 mM sodium acetate buffer pH 5.0 were mixed with 20 μL of bacterial supernatant and incubated 60 min at 37 °C [51]. The reactions were stopped by heating at 100 °C, 5 min before the analysis of products by DNS reagent. One unit (U) of activities (cellulase and polygalacturonase) was defined as the amount of enzyme releasing 1 μmol of reducing sugars (glucose)/min per 10^9^ bacterial cells.

For protease activity assay, 500 μL of 1% azocasein in 100 mM Tris HCl (pH 7.5) and 100 μL of culture supernatant were mixed and incubated 60 min at 37 °C and then 100 μL of 10% trichloroacetic acid were added to the reaction mixture. The sediment was removed by filtration and 500 μL of the supernatant were incubated with 166 μL of 1M NaOH 10 min at 25 °C and the absorbance was measured at 440 nm [52]. One unit (U) of the protease activity was defined as the amount of enzyme required to produce an absorbance change of 1.0/min per 10^9^ bacterial cells. All enzymatic activities were measured in three biological replicates using a РВ2201В spectrophotometer (SOLAR, Belarus).

### 4.7. Gene Expression Analysis

Plant leaves (one day after plant inoculation) were ground in liquid nitrogen in mortars. The obtained powder was resuspended in 1 mL of ExtractRNA Reagent (Evrogen, Moscow, Russia) and the subsequent procedures were performed according to the manufacturer’s instructions. Residual DNA was eliminated using a DNA-free kit (Life Technologies, Carlsbad, CA, USA). One microgram of DNAse-treated RNA was used for cDNA synthesis using RevertAid reverse transcriptase (Thermo Fisher Scientific, Waltham, MA, USA) according to the manufacturer’s instructions. Two microliters of 5-fold-diluted cDNA was used for qPCR. qPCR was performed using the EVA-Green-containing master mix (Syntol, Moscow, Russia) according to the manufacturer’s instructions.

Primers for target and reference genes (Table S1) were designed using Vector-NTI Version 9 software (Invitrogen, Waltham, MA, USA) and synthesized by Evrogen (Moscow, Russia). Genes encoding elongation factor 1-alpha and ATP-synthase subunit beta, the transcript level of which was confirmed by geNorm software (http://genorm.cmgg.be/) to be stable under the experimental conditions (data not shown), were used for normalization of the target gene expression. Relative expression levels were determined as the ratios between the quantities of cDNA corresponding to the target genes and values of the normalization factor, which was calculated for each sample using geNorm software based on transcript levels of reference genes. The presented data were obtained by the analysis of four-five biological replicates.

### 4.8. Determination of the Hydrogen Peroxide Level

H_2_O_2_-level in the tobacco leaves was measured 1 day after mock or bacterial inoculation. One day before the inoculation, plants were treated with water or salicylic acid as described above. H_2_O_2_-level was determined by the method based on the peroxide-mediated oxidation of Fe^2+^ followed by the reaction of Fe^3+^ with xylenol orange (Sigma, USA) [53]. Leaves (100 mg) were ground in 1 mL of cold 50 mM borate buffer, pH 8.4 in mortars. The homogenates were centrifuged (7000× g, 10 min) and 100 μL of the supernatants were added to 500 μL of the assay reagent (500 mM ammonium ferrous sulfate, 50 mM H_2_SO_4_, 200 mM xylenol orange and 200 mM sorbitol). The absorbance of the Fe^3+^-xylenol orange complex (*A*560) was detected after 45 min. Standard curves of H_2_O_2_ were obtained for each independent experiment by adding variable amounts of H_2_O_2_ to 100 mL of borate buffer mixed to 500 mL of assay reagent. Data were normalized and expressed as µmol H_2_O_2_ per gram of fresh weight. The presented data are means ± SD of three biological replicates.

## Data Availability

Not applicable.

## Supplementary Materials

The following are available online at https://www.mdpi.com/article/10.3390/ijms22179594/s1.
