# Supplementary material for: The Knockout of Enterobactin-Related Gene in Pectobacterium atrosepticum Results in Reduced Stress Resistance and Virulence towards the Primed Plants
Source: Int J Mol Sci. 2021 Sep 4;22(17):9594. doi: 10.3390/ijms22179594 (PMC8431002; doi:10.3390/ijms22179594)
Supplement: Supplementary file 1 [file ijms-22-09594-s001.zip › Figure_S1.pdf]

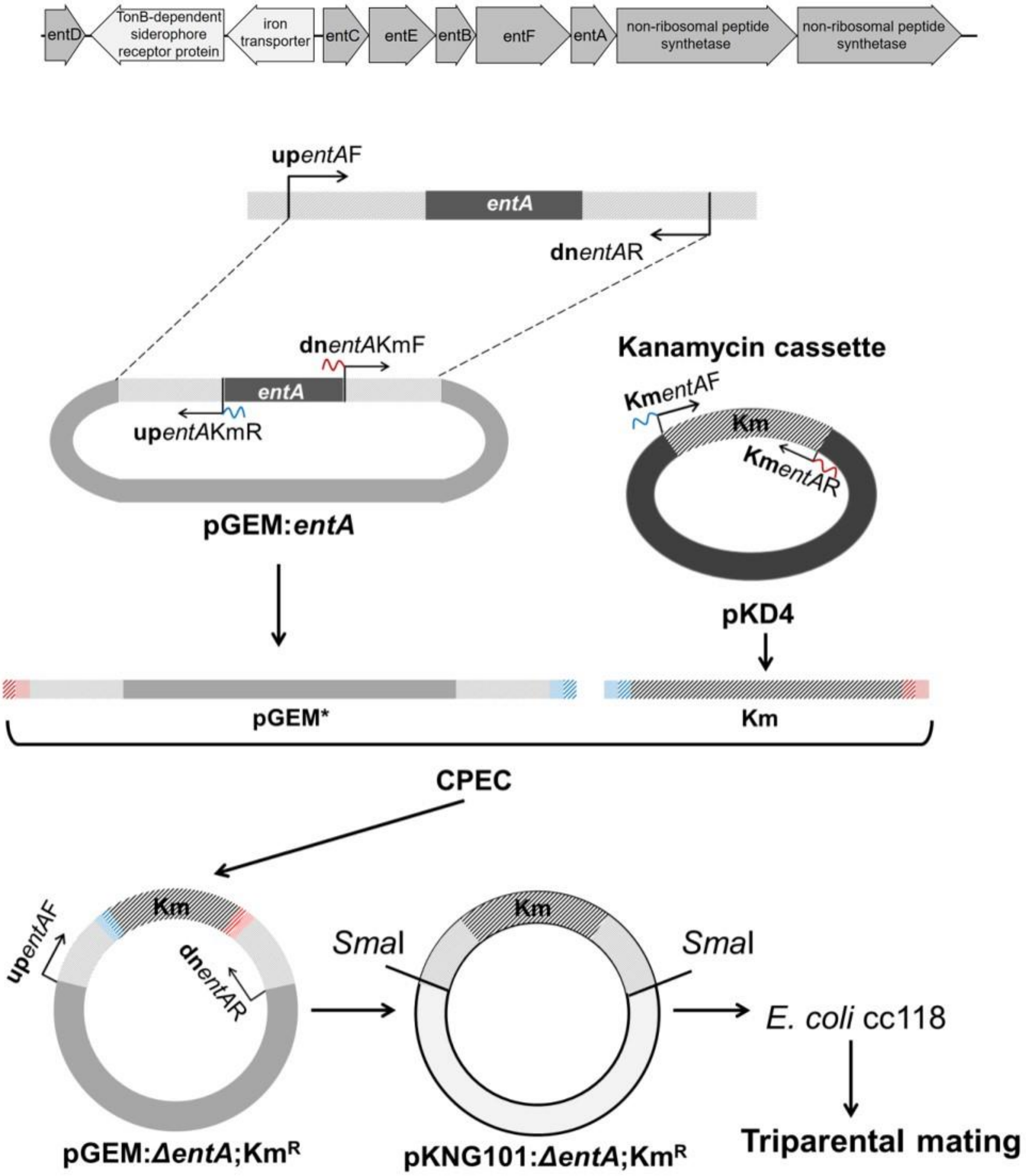

Figure S1. A scheme of the structure of the enterobactin gene cluster and the construction of entA deletion mutant (described in the text).
