# Supplementary material for: The Knockout of Enterobactin-Related Gene in Pectobacterium atrosepticum Results in Reduced Stress Resistance and Virulence towards the Primed Plants
Source: Int J Mol Sci. 2021 Sep 4;22(17):9594. doi: 10.3390/ijms22179594 (PMC8431002; doi:10.3390/ijms22179594)
Supplement: Supplementary file 1 [file ijms-22-09594-s001.zip › Table_S1.pdf]

Table S1. Bacterial strains, plasmids and primers used in this study.

Name	Description	Origin
**Strain**		
Pectobacterium atrosepticum SCRI1043 (*Pba*)	Wild type	[1]
Pectobacterium atrosepticum SCRI1043*ΔentA*	Mutant strain of SCRI1043 containing the kanamycin cassette in the chromosome; Km^R	This study
Pectobacterium atrosepticum SCRI1043comp*ΔentA*	Mutant strain of SCRI1043 containing the kanamycin cassette in the chromosome and the entA complementation construct on pGEM:entA; Amp^R	This study
Escherichia coli cc118	Host for suicidal vector pKNG101; *Δ(ara, leu) araD ΔlacX 74 galE galK PhoA20 thi-1 rpsE rpoB argE (am) recA1,* Sm^R	[2]
Escherichia coli HH26/pNJ5000	Mobilizing strain for conjugative transfer of the suicide vector pKNG101 into *Pba* cells; *tra+;* Tet^R	[3]
Escherichia coli NovaBlue	*endA 1hsdR17 (rK12–mK12+) supE44 thi-1 recA1 gyrA96 relA1 lacF′ [proA+ B+ lacIqZ ΔM15::Tn10 (*Tet^R*)]*	Novagen
**Plasmids**		
pGEM-T Easy	Linearized vector for cloning; *f1 ori* Amp^R *lacZ*	Promega
pGEM:*entA*	*f1 ori* Amp^R *lacZ entA*	This study
pGEM:*ΔentA*;Km^R	*f1 ori* Amp^R *lacZ* Km^R	This study
pKD4	Matrix for PCR amplification of kanamycin resistance cassette; *oriRγ rgnB bla* Km^R	[4]
pKNG101	Suicide mobilized vector for inactivation of target genes; *pir-ori R6K mobRK2 sacB* Sm^R	[5]
pKNG101:*ΔentA*;Km^R	Suicide plasmid carrying mutant locus *ΔentA*;Km^R; Km^R *SmR sacB*	This study
pGEM:*entA;* complementation construct	*f1 ori* Amp^R *lacZ entA*	This study

Primers

Primer name	Primer sequence 5'—3'
**Primers for mutagenesis**	
**up***entA*F	GCTGCGTACCGATGAAATGC
**dn***entA*R	CAATGTCTTTCTCGCCGCTG
**dn***entA*KmF	CCATGTCAGCCGTTAAGGGATGGCGGGGCAACGCTGACGGCCTGATTTG
**up***entA*KmR	CAGCTCCAGCCTACACAATCGAGGTTGTGCCTTGTTCATCATTTTC
**Km***entA*F	GATGAACAAGGCACAACCTCGATTGTGTAGGCTGGAGCTGCTTC
**Km***entA*R	GCCGTCAGCGTTGCCCCGCCATCCCTTAACGGCTGACATGGGAATTAGC

**Check***entA*F	CGAAAGGCGTGGAGATCGGC
**Check***entA*R	GCCAGCGGGGAGCCAATC
**Primers for complementation construct**	
promF	GCTTTCATCCATGACAGGCTCACATTTAG
**prom***entA*R	TGTGCCTTGTTCATCATAAGACAGCTCCTGCGCTCGCAAA
**prom***entA*F	TTTGCGAGCGCAGGAGCTGTCTTATGATGAACAAGGCACA
**entA***term*R	GATGCTGCCCTTTATCACTGTTCAGGCCGTCAGCGTTGCC
**entA***term*F	GGCAACGCTGACGGCCTGAACAGTGATAAAGGGCAGCATC
termR	CCTAAGCCTGCTATCTCCTCAGCATAATTTC
**Primers for qRT-PCR**	
NtAOCF	CGGCTCAACTGATTCCTCTACAAC
NtAOCR	GTTTGTTGCTAAAGGGGACAAGATC
NtLOXF	CACGCATAATGAGTTTGATAGTTTTG
NtLOXR	CTGAGGAGTTGGGTACTTAAATAGGC
NtEFF	GCCCAACACTTCTTGATGCTC
NtEFR	GACACCAGTTTCCACACGACC
NtATPF	GGTCGATGGCTTGGGAGTACC
NtATPR	GCACAGATTTACGTTCAATAATACCAG

1. Bell, K.S.; Sebaihia, M.; Pritchard, L.; Holden, M.T.G.; Hyman, L.J.; Holeva, M.C.; Thomson, N.R.; Bentley, S.D.; Churcher, L.J.C.; Mungall, K.; et al. Genome sequence of the enterobacterial phytopathogen *Erwinia carotovora* subsp. *atroseptica* and characterization of virulence factors. *Proc. Natl. Acad. Sci.* **2004**, *101*, 11105–11110.
2. Herrero, M.; de Lorenzo, V.; Timmis, K.N. Transposon vectors containing non-antibiotic resistance selection markers for cloning and stable chromosomal insertion of foreign genes in gram-negative bacteria. *J. Bacteriol.* **1990**, *172*, 6557–6567.
3. Grinter, N.J. A broad-host-range cloning vector transposable to various replicons. *Gene* **1983**, *21*, 133–143.
4. Datsenko, K.A.; Wanner, B.L. One-step inactivation of chromosomal genes in *Escherichia coli* K-12 using PCR products. *Proc. Natl. Acad. Sci.* **2000**, *97*, 6640–6645.
5. Kaniga, K.; Delor, I.; Cornelis, G.R. A wide-host-range suicide vector for improving reverse genetics in gram-negative bacteria: Inactivation of the *blaA* gene of *Yersinia enterocolitica*. *Gene* **1991**, *109*, 137–141.
